# Chemoradiation treatment patterns among United States Veteran Health Administration patients with unresectable stage III non-small cell lung cancer

**DOI:** 10.1186/s12885-021-08577-y

**Published:** 2021-07-16

**Authors:** Anna Hung, Kyung Min Lee, Julie A. Lynch, Yanhong Li, Pradeep Poonnen, Olga V. Efimova, Bradley J. Hintze, Trudy Buckingham, Candice Yong, Brian Seal, Michael J. Kelley, Shelby D. Reed

**Affiliations:** 1 Duke Clinical Research Institute, Duke University School of Medicine, Durham, NC USA; 2 Department of Population Health Sciences, Duke University School of Medicine, Durham, NC USA; 3 Department of Veteran Affairs Medical Center, Durham, NC USA; 4 Department of Veteran Affairs Salt Lake City Health Care System, Salt Lake City, UT USA; 5 Duke University Medical Center, Durham, NC USA; 6 AstraZeneca, Gaithersburg, MD USA

**Keywords:** Non-small cell lung Cancer, Concurrent and sequential Chemoradiation, Veterans health administration

## Abstract

**Background:**

The Veterans Health Administration (VHA) is the largest integrated health care system in the United States (US). Among VHA patients, the rate of use of concurrent chemoradiation therapy (CCRT) among those with unresectable, stage III non-small cell lung cancer (NSCLC) is unknown. The objective was to report recent CCRT treatment patterns in VHA patients and identify characteristics associated with receipt of CCRT.

**Methods:**

Using Department of Veteran Affairs (VA) Cancer Registry System data linked to VA electronic medical records, we determined rates of CCRT, sequential CRT (SCRT), radiation therapy (RT) only, chemotherapy (CT) only, and neither treatment.

**Results:**

Among 4054 VHA patients who met study criteria, CCRT rates slightly increased from 44 to 50% between 2013 and 2017. Factors associated with decreased odds of CCRT receipt compared to any other treatment included increasing age (adjusted odds ratio [aOR] per 10 years = 0.67; 95% CI: 0.60–0.76) and Charlson-Deyo comorbidity score (aOR = 0.94; 95% CI: 0.91–0.97). White race was associated with increased odds of CCRT receipt (aOR = 1.24; 95% CI: 1.004–1.53). In a chart review sample of 200 patients, less than half (*n* = 85) had a documented reason for not receiving CCRT. Among these, 29% declined treatment, and 71% did not receive CCRT due to “not being a candidate” for reasons related to frailty or lung nodules being too far apart for radiation therapy.

**Conclusions:**

CCRT rates among VHA patients with unresectable, stage III NSCLC slightly increased from 2013 to 2017; however in 2017, only half were receiving CCRT. Older patients and those with multiple comorbidities were less likely to receive CCRT and even when controlling for these factors, non-white patients were less likely to receive CCRT.

**Supplementary Information:**

The online version contains supplementary material available at 10.1186/s12885-021-08577-y.

## Introduction

In patients with unresectable stage III non-small cell lung cancer (NSCLC), high-level evidence from randomized controlled trials published starting in the 1990s have demonstrated that concurrent chemoradiation therapy (CCRT) results in improved overall survival compared to radiation therapy (RT) alone [1–4] or sequential chemoradiation therapy (SCRT) [5–9] with tolerable additional toxicity. However, guidelines also note that as part of the treatment selection process, one should consider a patient’s ability to tolerate CCRT [10]. For example, CCRT has a higher rate of grade 3 or 4 esophagitis than SCRT [5]. As a result, frail patients may not be able to tolerate CCRT [10–12].

In the United States (US), military veterans (i.e., those who served in the armed forces), are eligible for medical care from the Veterans Health Administration (VHA). The VHA is the largest integrated health care system in the US [13]. It has approximately 171 medical centers and 1112 outpatient sites of care and serves approximately 9 million patients each year [13]. The majority of VHA patients are male, married, white, and non-Hispanic [14]. Compared to the general US population, VHA patients tend to be older, have lower levels of income and education, and have a higher comorbidity burden [15]. In 2010, 18% of incident veteran cancer cases diagnosed in the VHA were lung cancer [16]. Many VHA patients are current (16%) or past (61%) smokers, which can impact histology and treatment of NSCLC [14].

Between 2001 and 2010, only one-quarter of VHA patients with stage III NSCLC received chemotherapy and radiation within 4 months of diagnosis and had unresectable disease [17]. Among those patients who received chemotherapy (CT) and RT, almost 60% received CCRT (as opposed to SCRT). The primary objective of this study was to report on more recent nationwide CCRT treatment patterns in VHA patients and identify patient- and facility-level factors associated with receipt of CCRT. The secondary objective was to report reasons why patients did not receive CCRT.

## Methods

### Cohort identification

VHA patients initially diagnosed with stage III NSCLC between January 1, 2013 and December 31, 2017 were identified using the VA Corporate Data Warehouse (CDW), which contains an extract of the VA Cancer Registry System (CRS). Patients were identified based on a primary cancer site of “lung/bronchus” in the VA CRS data and lung cancer diagnosis codes from the Ninth and Tenth Revisions of the International Classification of Diseases (Additional file 1: Appendix Table 1). Patients with NSCLC were retained based on histology codes (Additional file 1: Appendix Table 2). Patients were excluded if a stage IV NSCLC diagnosis was documented in VA CRS data within 1 month before or after the stage III diagnosis. This is because a documented diagnosis of stage IV before stage III was likely a medical record error, and a diagnosis of stage IV within 1 month after the diagnosis of stage III meant the patient would likely be treated for stage IV disease and CCRT would no longer be recommended.

VA CDW data were used to exclude patients who underwent lung resection in the 180 days following diagnosis (see procedure codes in Additional file 1: Appendix Tables 3a, 3b, and 3c) to identify those with unresectable stage III disease. To further limit the study population to patients who were receiving cancer care within the VHA, only patients who had at least two visits for cancer care in the 120 days following diagnosis were included. To ensure these visits were related to cancer care, patients had to have at least two visits to clinics related to cancer care based on clinic ‘stop’ codes (Additional file 1: Appendix Table 4) or at least two clinical notes that mentioned ‘lung cancer’. Next, National Death Index data were used to exclude patients who died within 45 days of diagnosis to avoid a bias towards underestimating treatment rates. Lastly, to account for receipt of chemotherapy and/or radiotherapy in non-VHA settings documented by local cancer registrars, only patients whose VA CRS abstract status was complete were included.

### Treatment definitions

We based our initial treatment definitions on a previous VA study, examining CT and RT within 120 days of diagnosis [17]. Chemotherapy (CT), based on the drugs reported in Additional file 1: Appendix Table 5, and RT, based on the procedure codes in Additional file 1: Appendix Table 6, that occurred within 120 days of diagnosis were identified. “CCRT” was defined as CT and RT that started within 14 days of each other. “SCRT” was defined as the receipt of CT and RT within 120 days of diagnosis that did not start within 14 days of each other. “CT only” was defined as CT alone within 120 days of diagnosis, and “RT only” was defined as RT alone within 120 days of diagnosis. “Neither treatment” was defined as not having received CT or RT within 120 days of diagnosis.

To assess the accuracy of applying these definitions to data from the VA CRS and CDW, 200 charts were randomly selected for review to assess concordance. Among those who did not receive CCRT, reasons for not receiving CCRT, if documented, were abstracted.

We performed three sensitivity analyses. First, we lengthened the time window used to define initial treatment from 120 days to 180 days of cancer diagnosis. Second, we varied the 14-day time window to define CCRT to 7 days, 21 days, and 30 days, since past studies have used different time windows [17, 18]. Third, given concerns about underreporting of radiation therapy services received outside of VHA, we narrowed the cohort to patients receiving care at VHA facilities equipped to provide on-site radiation therapy services.

### Patient- and facility-level characteristics

Patient- and facility-level characteristics were derived from CDW and VA CRS data corresponding to the initial cancer diagnosis year unless otherwise noted. These included sociodemographic characteristics such as age, gender, race, ethnicity, marital status, Medicare enrollment at any point between 2013 and 2017, Medicaid eligibility, VHA priority status, rurality of patient residence, distance between patient residence and VA medical center, histology, smoking status, and Charlson-Deyo comorbidity score. In the US, Medicare and Medicaid are other health insurance programs generally for older adults and those with lower incomes, respectively [19, 20]. Since VHA patients can also have additional insurance coverage such as through Medicare and Medicaid, it is important to account for these characteristics since additional insurance coverage can affect treatment. VHA priority groups are also important to account for when examining VHA care because they determine copay (i.e., costs that patients pay for health care) levels and how soon after military service patients are eligible for health care benefits, which can also affect treatment [21, 22]. Generally, a disability that is highly connected to military service (e.g., ≥50%) leads to assignment to a higher priority group with no copay requirements [21]. Other factors also impact priority group assignment. Such factors include income level and military service during specific time periods [21].

In addition to patient-level characteristics, facility-level characteristics were examined. These included whether the medical center had been certified by Commission on Cancer, geographic region, total number of unique patients seen at oncology clinics, and total number of oncologists at the medical center. In the US, the Commission on Cancer establishes standards to ensure quality, multidisciplinary, and comprehensive cancer care delivery in health care settings and has certified more than 1500 programs [23]. Geographic regions were measured as Census regions, which are regions set by the United States Census Bureau framework for grouping states to allow for consistency across time and studies [24]. The aforementioned patient- and facility-level characteristics were examined and adjusted for in models because all could be associated with receipt of CCRT.

### Statistical analysis

Descriptive statistics were provided for receipt of CCRT, SCRT, RT only, CT only, and neither treatment overall, by calendar year, and by Census region. In addition, variations in treatment patterns were also examined across VHA facilities. Bivariate associations between a given patient or facility characteristic and the treatment type were analyzed using Chi-square tests for categorical variables and Kruskal-Wallis tests for continuous variables.

Generalized linear mixed models using binomial distributions and logit links and accounting for clustering by VA facility were used to identify factors independently associated with CCRT receipt compared to other treatment (SCRT, CT only, or RT only). Statistical significance was defined at an alpha of 0.05. All analyses were performed in SAS 9.2 (Cary, NC).

The study was approved by the institutional review boards at Duke University, Durham VA, and VA Salt Lake City.

## Results

### Study population

**Fig. 1:**
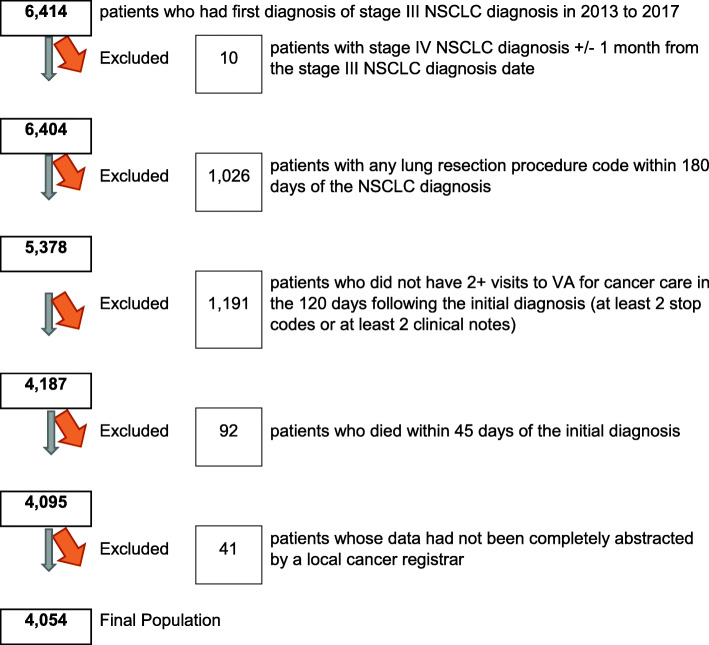
Cohort identification

Between January 1, 2013 and December 31, 2017, we identified 6414 VHA patients with stage III NSCLC. After excluding those with stage IV disease within 1 month of stage III diagnosis (*n* = 10), then those who underwent a lung resection procedure (*n* = 1026), then those who were not receiving their cancer care within VHA (*n* = 1191), then those who died within 45 days (*n* = 92), and then those who had incomplete registry records (*n* = 41), our final study population consisted of 4054 patients (Fig. 1).

### Treatment rates

**Fig. 2:**
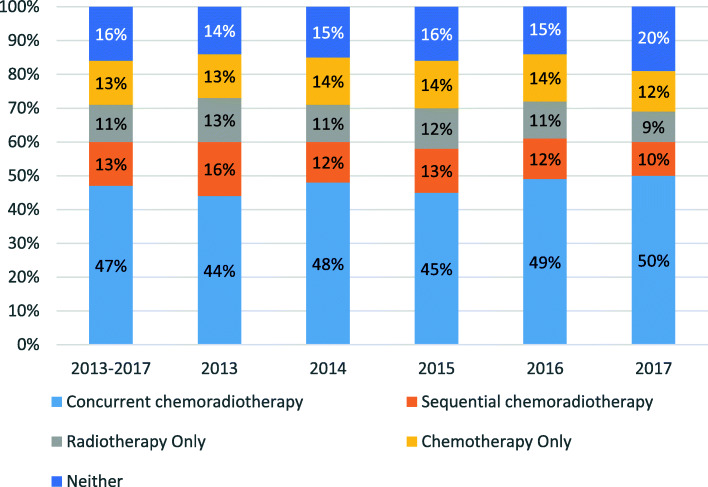
Total and annual treatment rates in the full study population (*n =* 4054)

**Fig. 3:**
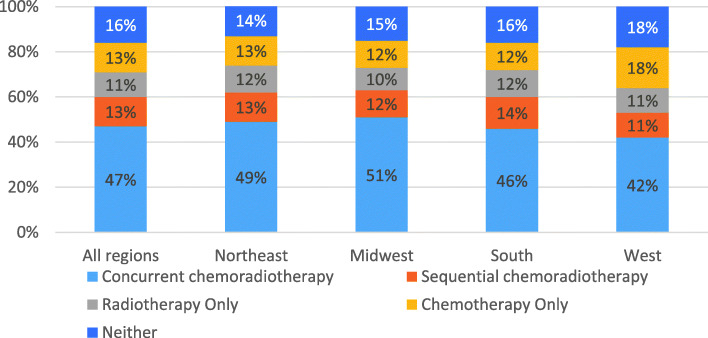
Treatment rates by geographic region in the full study population (*n =* 4054)

**Fig. 4:**
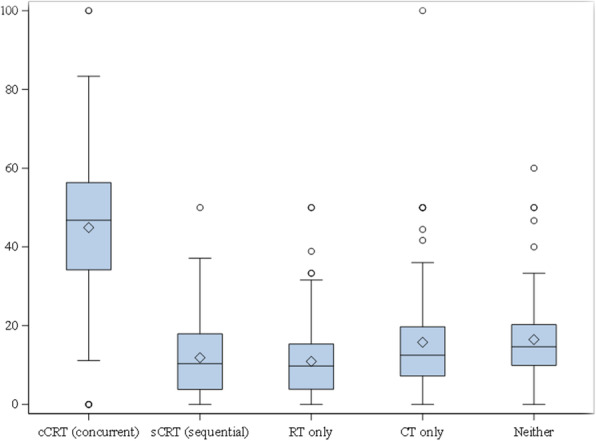
Variation in treatment rates across the 108 medical centers for the full study population

Forty-seven percent of patients received CCRT (Fig. 2). This rate fluctuated between 44 and 50% annually between 2013 and 2017, and between 42 and 51% across geographic regions (Figs. 2 and 3). The rate also varied by medical center. Across the 108 medical centers, the mean and median CCRT rates were 45 and 47%, respectively, with an interquartile range of 34 to 56% (Fig. 4, Additional file 1: Appendix Table 7). Rates of SCRT, RT only, CT only, and neither treatment varied between 10 and 20% overall, as well as by year and geographic region (Figs. 2 and 3). Similarly, mean and median rates of SCRT, RT only, CT only, and neither treatment across the medical centers varied between 10 and 16% (Fig. 4, Additional file 1: Appendix Table 7).

In our chart review of 200 randomly selected records, we found 90% concordance between our use of structured data to identify treatments received versus manual review employing the same treatment definitions. Sensitivity analyses showed that treatment rates minimally changed when extending the initial treatment time window from 120 days to 180 days (Additional file 1: Appendix Table 8), differed more when varying the CCRT definition as CT and RT within 14 days to 7, 21, and 30 days (Additional file 1: Appendix Table 9), and minimally changed when restricting the population to patients who were seen at medical centers that provided on-site radiation therapy services (Additional file 1: Appendix Figs. 1–3).

### Patient and facility characteristics associated with receipt of CCRT

The overall study population had a mean age of 68.7 years, and was predominantly male (97.5%), white (78.0%), and non-Hispanic (94.4%; Table 1). The majority were enrolled in Medicare (82.9%), few were eligible for Medicaid (1.7%), and the mean Charlson-Deyo comorbidity score was 3.4. Approximately half had a non-service connected disability (49.9%), were married (44.6%), and lived in an urban setting (62.4%), with a median distance between their residence and the medical center of 66.3 miles. Close to half of patients (51.9%) had squamous cancer, and most were current (56.0%) or former (34.5%) smokers. Almost three-quarters (72.2%) were seen at a medical center that was certified by Commission on Cancer, 44.5% were seen in the South, and the median numbers of oncology patients seen and oncologists working at medical centers during the diagnosis year were 196.0 and 12.0, respectively.

**Table 1:** Patient and medical center characteristics in all veterans and by treatment group

***Characteristics***	***All patients (N = 4054)***	***CCRT (N = 1893)***	***SCRT (N = 514)***	***RT only (N = 466)***	***CT only (N = 541)***	***Neither (N = 640)***	***P-value*** ^***a***^
**Patient-related**							
**Age**							
Mean (SD)	68.7 (8.2)	67.3 (7.4)	67.4 (7.3)	72.3 (8.9)	69.0 (8.4)	71.4 (8.9)	
Median (Q1-Q3)	68.0 (63.0–74.0)	67.0 (63.0–71.0)	67.0 (63.0–71.0)	71.0 (66.0–80.0)	67.0 (63.0–75.0)	70.0 (65.0–78.0)	<.0001
**Gender**							
Male	3954 (97.5%)	1847 (97.6%)	496 (96.5%)	457 (98.1%)	530 (98.0%)	624 (97.5%)	0.5120
**Race**							
White	3164 (78.0%)	1499 (79.2%)	392 (76.3%)	343 (73.6%)	443 (81.9%)	487 (76.1%)	0.0053
Black or African American	669 (16.5%)	304 (16.1%)	90 (17.5%)	88 (18.9%)	68 (12.6%)	119 (18.6%)	
Other	67 (1.7%)	21 (1.1%)	15 (2.9%)	+	13 (2.4%)	+	
Unknown	154 (3.8%)	69 (3.6%)	17 (3.3%)	++	17 (3.1%)	++	
**Ethnicity**							
Hispanic or Latino	97 (2.4%)	36 (1.9%)	13 (2.5%)	11 (2.4%)	22 (4.1%)	15 (2.3%)	0.0310
Not Hispanic or Latino	3826 (94.4%)	1803 (95.2%)	490 (95.3%)	440 (94.4%)	493 (91.1%)	600 (93.8%)	
Unknown	131 (3.2%)	54 (2.9%)	11 (2.1%)	15 (3.2%)	26 (4.8%)	25 (3.9%)	
**Marital Status** ^**b**^							
Married	1810 (44.6%)	++	++	++	++	++	0.2897
Not Married	2228 (55.0%)	1014 (53.6%)	281 (54.7%)	259 (55.6%)	290 (53.6%)	384 (60.0%)	
Unknown	16 (0.4%)	+	+	+	+	+	
**Medicare enrollment any time between 2013 and 2017**							
Yes	3361 (82.9%)	1522 (80.4%)	426 (82.9%)	414 (88.8%)	441 (81.5%)	558 (87.2%)	<.0001
**Medicaid-eligible**							
Yes	68 (1.7%)	27 (1.4%)	+	+	++	+	0.4563
No	3910 (96.4%)	1835 (96.9%)	491 (95.5%)	448 (96.1%)	517 (95.6%)	619 (96.7%)	
Unknown	76 (1.9%)	31 (1.6%)	++	++	+	++	
**Priority status**							
Non-service connected	2021 (49.9%)	917 (48.4%)	251 (48.8%)	262 (56.29%)	258 (47.7%)	333 (52.0%)	0.0553
Service connected < 50%	596 (14.7%)	269 (14.2%)	77 (15.0%)	68 (14.6%)	85 (15.7%)	97 (15.2%)	
Service connected 50 to 100%	1437 (35.4%)	707 (37.3%)	186 (36.2%)	136 (29.2%)	198 (36.6%)	210 (32.8%)	
**Rurality of patient residence**							
Urban	2531 (62.4%)	1178 (62.2%)	319 (62.1%)	310 (66.5%)	318 (58.8%)	406 (63.4%)	0.3908
**Distance between patient residence and medical center**							
Mean (SD)	93.2 (122.4)	85.9 (85.9)	88.6 (143.3)	83.9 (76.1)	114.5 (185.2)	107.2 (150.5)	
Median (Q1-Q3)	66.3 (20.6–127.5)	64.4 (20.5–122.1)	56.9 (18.6–113.6)	66.1 (18.2–130.0)	71.7 (27.0–131.4)	74.4 (21.2–145.4)	0.0061
Unknown	+	+	+	+	+	+	
**Histology**							
Squamous	2102 (51.9%)	1027 (54.3%)	291 (56.6%)	260 (55.8%)	229 (42.3%)	295 (46.1%)	<.0001
Adenocarcinoma	1447 (35.7%)	656 (34.7%)	171 (33.3%)	141 (30.3%)	236 (43.6%)	243 (38.0%)	
Other	505 (12.5%)	210 (11.1%)	52 (10.1%)	65 (13.9%)	76 (14.0%)	102 (15.9%)	
**Smoking status**							
Current smoker	2272 (56.0%)	1127 (59.5%)	299 (58.2%)	243 (52.2%)	266 (49.2%)	337 (52.7%)	0.0003
Former smoker	1400 (34.5%)	619 (32.7%)	165 (32.1%)	176 (37.8%)	202 (37.3%)	238 (37.2%)	
Never smoker	133 (3.3%)	47 (2.5%)	15 (2.9%)	17 (3.7%)	29 (5.4%)	25 (3.9%)	
Unknown	249 (6.1%)	100 (5.3%)	35 (6.8%)	30 (6.4%)	44 (8.1%)	40 (6.3%)	
**Charlson-Deyo comorbidity score**							
Mean (SD)	3.4 (2.6)	3.2 (2.4)	3.3 (2.6)	4.0 (2.9)	3.3 (2.6)	3.6 (2.6)	
Median (Q1-Q3)	3.0 (1.0–5.0)	3.0 (1.0–4.0)	3.0 (2.0–4.0)	3.0 (2.0–6.0)	3.0 (1.0–4.0)	3.0 (2.0–5.0)	<.0001
0	333 (8.2%)	167 (8.8%)	++	++	++	++	<.0001
1	684 (16.9%)	352 (18.6%)	76 (14.8%)	61 (13.1%)	83 (15.3%)	112 (17.5%)	
2	676 (16.7%)	315 (16.6%)	94 (18.3%)	67 (14.4%)	106 (19.6%)	94 (14.7%)	
3	760 (18.7%)	366 (19.3%)	109 (21.2%)	80 (17.2%)	96 (17.7%)	109 (17.0%)	
4+	1565 (38.6%)	675 (35.7%)	185 (36.0%)	225 (48.3%)	197 (36.4%)	283 (44.2%)	
Unknown	36 (0.9%)	18 (1.0%)	+	+	+	+	
**Medical Center-related**							
**Certified by Commission on Cancer**							
Yes	2928 (72.2%)	1379 (72.8%)	387 (75.3%)	354 (76.0%)	376 (69.5%)	432 (67.5%)	0.0162
No	1107 (27.3%)	503 (26.6%)	++	++	++	++	
Unknown	19 (0.5%)	11 (0.6%)	+	+	+	+	
**Geographic (Census) region**							
Northeast	530 (13.1%)	++	++	++	++	++	<.0001
Midwest	1068 (26.3%)	542 (28.6%)	128 (24.9%)	110 (23.6%)	130 (24.0%)	158 (24.7%)	
South	1806 (44.5%)	829 (43.8%)	245 (47.7%)	225 (48.3%)	221 (40.9%)	286 (44.7%)	
West	622 (15.3%)	261 (13.8%)	69 (13.4%)	70 (15.0%)	110 (20.3%)	112 (17.5%)	
Puerto Rico	28 (0.7%)	+	+	+	+	+	
**Total number of unique patients seen at oncology clinics at medical center**							
Mean (SD)	217.7 (157.2)	226.0 (159.5)	218.2 (157.5)	204.1 (149.9)	196.5 (150.4)	220.3 (158.9)	
Median (Q1-Q3)	196.0 (95.0–302.0)	208.0 (99.0–328.0)	191.0 (93.0–301.0)	185.5 (90.0–290.0)	170.0 (85.0–275.0)	204.0 (93.5–318.5)	0.0009
**Total number of oncologists at medical center**							
Mean (SD)	12.8 (7.7)	10.6 (5.9)	10.8 (5.2)	10.9 (5.5)	9.6 (5.8)	9.7 (5.7)	
Median (Q1-Q3)	12.0 (7.0–17.0)	11.0 (6.0–15.0)	11.0 (6.0–14.0)	11.0 (7.0–15.0)	9.0 (5.0–14.0)	9.0 (5.0–14.0)	<.0001

+ Cell size < 11 suppressed per VA policy

++ Suppressed cell size so one cannot calculate sample size for cells with *n* < 11

^a^*P*-value based on Chi-square tests for categorical variables and Kruskal-Wallis tests for continuous variables

^b^Single or unknown marriage status not shown due to small cell sizes

Among the 3414 patients who received treatment (i.e., CCRT, SCRT, RT only, or CT only), factors associated with increased odds of receipt of CCRT compared to any other treatment included white race (adjusted OR [aOR] = 1.24; 95% CI: 1.00–1.53) and later diagnosis year (2017 vs 2013: aOR = 1.65; 95% CI: 1.27–2.16; 2016 vs 2013: aOR = 1.36; 95% CI: 1.06–1.76). Factors associated with decreased odds of CCRT receipt compared to any other treatment included increasing age (aOR per 10 years = 0.67; 95% CI: 0.60–0.76) and Charlson-Deyo comorbidity score (aOR = 0.94; 95% CI: 0.91–0.97; Table 2).

**Table 2:** Adjusted odds ratios for receipt of concurrent CRT versus any other treatment ^a^

Characteristic	Estimate	95% Confidence Limits	
Patient-related		Lower	Upper
Female	0.64	0.40	1.03
**White**	**1.24**	**1.004**	**1.53**
Hispanic	0.71	0.39	1.31
Married	1.13	0.96	1.33
Medicare enrollment any time between 2013 and 2017	1.13	0.89	1.43
Medicaid-eligible	0.63	0.34	1.14
Priority status (reference: non-service connected)			
Service connected ≥50%	1.12	0.94	1.33
Service connected < 50%	1.01	0.81	1.27
Urban residence	1.09	0.91	1.33
Histology (reference: squamous)			
Adenocarcinoma	0.91	0.77	1.07
Other	0.80	0.62	1.02
Smoking status (reference: never)			
Current smoker	1.41	0.90	2.20
Former smoker	1.34	0.85	2.10
Unknown	1.13	0.65	1.95
Diagnosis year (reference: 2013)			
**2017**	**1.65**	**1.27**	**2.16**
**2016**	**1.36**	**1.06**	**1.76**
2015	1.11	0.88	1.39
2014	1.22	0.98	1.53
**Age (increment = 10)**	**0.67**	**0.60**	**0.76**
**Charlson-Deyo comorbidity score (increment = 1)**	**0.94**	**0.91**	**0.97**
Distance between patient residence and medical center based on zip code (increment = 100 miles)	0.95	0.84	1.08
**Medical center-related**			
Geographic (Census) region (reference: South)			
Northeast	1.01	0.66	1.55
Midwest	1.19	0.82	1.73
West	0.89	0.60	1.33
Medical Center Certified by Commission on Cancer	0.93	0.68	1.27
Total number of unique patients seen at oncology clinics at medical center of diagnosis during diagnosis year (increment = 100)	1.03	0.92	1.15
Total number of oncologists at medical center of diagnosis (increment = 10)	0.93	0.70	1.22

^a^Final model based on 3093 patients who had no missing data across variables. Those from Puerto Rico were excluded due to small sample size

### Chart review and reasons for not receiving CCRT

Among the 200 charts reviewed, 142 patients did not receive CCRT. Of these, no reason was documented in electronic medical records for 57 patients (40%; Additional file 1: Appendix Table 10). Of the 85 patients with a documented reason, 25 (29%) patients declined treatment and 60 (71%) were not considered candidates for CCRT. Of these, 40% were documented as too frail and 23% were considered to have disease that was too extensive or had lung nodules too far apart for radiation therapy. The remainder had no further information provided or were not a candidate due to comorbidities, disease progression/multiple cancers, or age (cell sizes too small to report).

## Discussion

This study documents the most recent CCRT treatment patterns in a national cohort of VHA patients with unresectable stage III NSCLC in the United States. Our study found that among this cohort, the proportion of patients receiving CCRT increased slightly from 44% in 2013 to 50% in 2017. A previous study found that from 2001 to 2010, 59% of VHA patients who received RT and CT within 4 months of being diagnosed with unresectable, stage III NSCLC received CCRT as opposed to SCRT [13]. Our analysis of the 2013–2017 data showed that this proportion increased to 79%, demonstrating that prescribers are following the increasing evidence that CCRT is more efficacious than SCRT [5–10].

However, among the whole cohort of VHA patients with unresectable stage III NSCLC, less than half (47%) are receiving CCRT. After excluding those who received no treatment, approximately 55% received CCRT, which is similar to the 52% that was recently reported to have received CCRT in a US Medicare population (generally at least 65 years of age and above) based on 2009 to 2014 data [18]. These rates are higher than reported rates outside the US. A study in China using 2013 to 2017 data reported CCRT use in 45% of 749 patients with unresectable stage III NSCLC treated at a single institution [25]. In the Netherlands, a multicenter retrospective study found that among 216 patients at least 70 years of age diagnosed with unresectable stage III NSCLC between 2009 and 2013, 33% received CCRT [26]. In Turkey, a single-hospital study examining 130 patients at least 70 years of age with unresectable stage III NSCLC over 2005 to 2017 found that CCRT was used in 23% of patients [27].

Our results should be interpreted with an understanding of the VHA patient population, which is more likely to smoke currently or formerly compared to the general US population [28]. In our study cohort, 91% were current (56%) or former smokers (35%), which was most similar to the Netherlands cohort (40% current and 54% former smokers) [26]. The high rate of smoking may be concerning since studies in stage III NSCLC patients indicate that current smoking is associated with poorer prognosis [29–31]. A recent study found that high-risk smokers (i.e., current or former smokers with a 30 or more pack-year smoking history) with lung cancer had shorter survival and poorer pulmonary function, as well as were more likely to have squamous histology [32]. In our study, 52% of patients had squamous histology which is higher than the 42% reported in the US Medicare population cohort. It is also higher than the 32 and 46% reported for the study cohorts in the Netherlands and Turkey, but lower than the 55% reported for the study cohort in China [25–27]. In our study, neither smoking status nor squamous histology was associated with receipt of CCRT after adjusting for other patient- and facility-level factors.

Patient factors that were associated with not receiving CCRT included increasing age and comorbidity burden, consistent with studies in US Medicare populations [18]. Chart review revealed that the majority of documented reasons for patients not receiving CCRT were related to not being a candidate due to frailty. This is aligned with a recent survey of US oncologists in which the most reported reason (64%) for not recommending CCRT was that the patient would be unlikely to tolerate due to comorbidities, poor performance status, and/or advanced age [33]. Other reported reasons included patient preference (47%), targetable mutation identified in the patient (40%), ability of the patient to travel consistently to receive treatment (40%), and cost (34%). Similar to the number one reported reason, the most common motive in the Netherlands for not receiving CCRT was comorbidity and/or performance status (58%) [26].

Guidelines agree that a patient’s anticipated tolerance to therapy is an important factor when selecting treatment; however, studies have suggested that age alone is an insufficient reason to forego CCRT [10, 34, 35]. For example, a phase 3 randomized trial and its long-termfollow-up study assessed CCRT using low-dose carboplatin versus RT only in elderly patients (> 70 years) with unresectable NSCLC and found that in both the short-term and long-term, CCRT increased overall survival [35]. Real-world studies from the Netherlands and Turkey also suggest that CCRT use in older patients (70 years of age and above) is associated with better survival [26, 27]. However, a study of VHA patients with NSCLC found that advancing age was a much stronger negative predictor of treatment receipt than comorbidity, contrary to evidence and guidelines stressing the importance of assessing comorbidity.

In addition to age and comorbidity burden, there were also differences in CCRT rates by race. In adjusted analyses, white patients were modestly more likely to receive CCRT compared to non-white patients. This is consistent with studies in US non-veteran populations that found that blacks, Asian-Pacific Islanders, and Hispanics were less likely receive CCRT compared to whites [18, 36]. Future studies should be performed to better understand these potential racial disparities and reasons for non-receipt of CCRT so that effective interventions can be developed to address barriers to NCCN recommended care.

### Limitations

Findings from this study should be interpreted in light of the limitations. We assessed treatment patterns in patients with unresectable, stage III NSCLC who were receiving their cancer care within VHA. Our results are not generalizable beyond this sample. Our treatment definitions were based on chemotherapy and radiation therapy start dates because the VA CRS did not systematically collect more detailed information regarding chemotherapy and radiation therapy administration for all patients. For example, if patients received their chemotherapy in VHA, but their radiation therapy in a non-VHA setting, only documentation of chemotherapy and radiation therapy start dates was required. However, we used previously published treatment definitions and performed multiple sensitivity analyses (e.g., varying time windows, examining rates among VHA facilities equipped to provide on-site radiation therapy services) to ensure that our estimates were robust. Additionally, our study period did not extend beyond 2017 because this was the most recent data available at the start of the study, given the time required for cancer registrars to carefully review and abstract data for the VA CRS. Our data also did not provide Eastern Cooperative Oncology Group performance status for all patients. Therefore, we calculated and adjusted for Charlson-Deyo comorbidity score, which could be determined for all patients.

## Conclusion

CCRT rates among VHA patients with unresectable, stage III NSCLC slightly increased from 2013 to 2017; however in 2017, only half were receiving CCRT. Negative predictors included increasing age and comorbidity burden, while positive predictors included white race. Providers should consider comorbidity burden in addition to age, and be aware of potential racial disparities, when selecting among various therapeutic options. In chart review, reasons for patients not receiving CCRT were documented in less than half of patients. More research is needed to understand why eligible patients do not receive CCRT.

## Supplementary Information

**Additional file 1: Appendix.** International Classification of Diseases and Related Health Problems Codes for Lung Cancer. **Appendix Table 2**. Histology Codes for Non-small Cell Lung Cancer. Appendix Table 3a. CPT Codes for Lung Resection. **Appendix Table 3b**. ICD-9 Procedure Codes for Lung Resection. **Appendix Table 3c**. ICD-10 Procedure Codes for Lung Resection. **Appendix Table 4**. Stop Codes for VA Oncology Clinics. **Appendix Table 5**. Chemotherapy Drugs. **Appendix Table 6**. Radiation Therapy Codes. **Appendix Table 7**. Variation in Treatment Rates Across 108 Medical Centers. **Appendix Table 8**. Treatment Rates Based on Expanding Initial Treatment Time Window from 120 days to 180 days. **Appendix Table 9**. Treatment Rates Based on Varying Time Window Between CT and RT to Define CCRT. **Appendix Table 10**. Reasons for Not Receiving CCRT per Chart Review. **Appendix Fig. 1**. Total and Annual Treatment Rates in the Subgroup Seen at Medical Centers with Radiation Therapy (*n* = 2067). **Appendix Fig. 2**. Treatment Rates by Census Region in the Subgroup Seen at Medical Centers with Radiation Therapy (*n =* 2067). **Appendix Fig. 3**. Boxplot of Variation in Treatment Rates Across 38* Medical Centers that Provide Radiation Therapy.

## Data Availability

Data analyzed in the current study are not publicly available because they contain protected health information. These data can be made available from the corresponding author to credentialed VA clinicians and researchers on a written request and in accordance with VA regulations.

## Abbreviations

aOR: Adjusted odds ratio
CDW: Corporate data warehouse
CRS: Cancer registry system
CCRT: Concurrent chemoradiation therapy
CI: Confidence interval
CT: Chemotherapy
NSCLC: Non-small cell lung cancer
RT: Radiation therapy
SCRT: Sequential chemoradiation therapy
US: United States
VA: Department of Veterans Affairs
VHA: Veterans Health Administration
