# Supplementary material for: Chemoradiation treatment patterns among United States Veteran Health Administration patients with unresectable stage III non-small cell lung cancer
Source: BMC Cancer. 2021 Jul 16;21:824. doi: 10.1186/s12885-021-08577-y (PMC8285779; doi:10.1186/s12885-021-08577-y)
Supplement: Supplementary file 1 — Additional file 1: Appendix. International Classification of Diseases and Related Health Problems Codes for Lung Cancer. Appendix Table 2. Histology Codes for Non-small Cell Lung Cancer. Appendix Table 3a. CPT Codes for Lung Resection. Appendix Table 3b. ICD-9 Procedure Codes for Lung Resection. Appendix Table 3c. ICD-10 Procedure Codes for Lung Resection. Appendix Table 4. Stop Codes for VA Oncology Clinics. Appendix Table 5. Chemotherapy Drugs. Appendix Table 6. Radiation Therapy Codes. Appendix Table 7. Variation in Treatment Rates Across 108 Medical Centers. Appendix Table 8. Treatment Rates Based on Expanding Initial Treatment Time Window from 120 days to 180 days. Appendix Table 9. Treatment Rates Based on Varying Time Window Between CT and RT to Define CCRT. Appendix Table 10. Reasons for Not Receiving CCRT per Chart Review. Appendix Fig. 1. Total and Annual Treatment Rates in the Subgroup Seen at Medical Centers with Radiation Therapy (n = 2067). Appendix Fig. 2. Treatment Rates by Census Region in the Subgroup Seen at Medical Centers with Radiation Therapy (n = 2067). Appendix Fig. 3. Boxplot of Variation in Treatment Rates Across 38* Medical Centers that Provide Radiation Therapy. [file 12885_2021_8577_MOESM1_ESM.docx]

**APPENDIX**

**Appendix Table 1. International Classification of Diseases and Related Health Problems Codes for Lung Cancer**

| **Code** | **Description** |
| --- | --- |
| **ICD-9** |  |
| 162.0 | Malignant neoplasm of trachea bronchus and lung |
| 162.1 | Malignant neoplasm of trachea |
| 162.2 | Malignant neoplasm of main bronchus |
| 162.3 | Malignant neoplasm of upper lobe, bronchus or lung |
| 162.4 | Malignant neoplasm of middle lobe, bronchus or lung |
| 162.5 | Malignant neoplasm of lower lobe, bronchus or lung |
| 162.8 | Malignant neoplasm of other parts of bronchus or lung |
| 162.9 | Malignant neoplasm of bronchus and lung, unspecified |
| **ICD-10** |  |
| C34.0x | Malignant neoplasm of main bronchus |
| C34.1x | Malignant neoplasm of upper lobe, bronchus or lung |
| C34.2x | Malignant neoplasm of middle lobe, bronchus or lung |
| C34.3x | Malignant neoplasm of lower lobe, bronchus or lung |
| C34.8x | Malignant neoplasm of overlapping site of bronchus and lung |
| C34.9x | Malignant neoplasm of unspecified part of bronchus or lung |

**Appendix Table 2. Histology Codes for Non-small Cell Lung Cancer**

| **Histology Code** | **Description** |
| --- | --- |
| 80000 | Neoplasm, benign |
| 80001 | Neoplasm, uncertain whether benign or malignant |
| 80003 | Neoplasm, malignant |
| 80013 | Tumor cells, malignant |
| 80023 | Malignant tumor, small cell type |
| 80033 | Malignant tumor, giant cell type |
| 80043 | Malignant tumor, spindle cell type |
| 80102 | Carcinoma in situ, NOS |
| 80103 | Carcinoma, NOS |
| 80113 | Epithelioma, malignant |
| 80123 | Large cell carcinoma, NOS |
| 80133 | Large cell neuroendocrine carcinoma |
| 80143 | Large cell carcinoma with rhabdoid phenotype |
| 80203 | Carcinoma, undifferentiated type, NOS |
| 80213 | Carcinoma, anaplastic type, NOS |
| 80223 | Pleomorphic carcinoma |
| 80303 | Giant cell and spindle cell carcinoma |
| 80313 | Giant cell carcinoma |
| 80323 | Spindle cell carcinoma |
| 80333 | Pseudosarcomatous carcinoma |
| 80462 | Non-small-cell carcinoma, in situ |
| 80463 | Non-small cell carcinoma |
| 80502 | Papillary carcinoma in situ |
| 80503 | Papillary carcinoma, NOS |
| 80522 | Papillary squamous cell carcinoma, non-invasive |
| 80523 | Papillary squamous cell carcinoma |
| 80702 | Squamous cell carcinoma in situ, NOS |
| 80703 | Squamous cell carcinoma, NOS |
| 80713 | Sq. cell carcinoma, keratinizing, NOS |
| 80723 | Sq. cell carcinoma, lg. cell, non-ker. |
| 80732 | Squamous cell carcinoma, in situ |
| 80733 | Sq. cell carcinoma, sm. cell, non-ker. |
| 80743 | Sq. cell carcinoma, spindle cell |
| 80753 | Squamous cell carcinoma, adenoid |
| 80762 | Sq. cell carc. in situ with question. stromal invas. |
| 80763 | Sq. cell carcinoma, micro-invasive |
| 80783 | Squamous cell carcinoma with horn formation |
| 81402 | Adenocarcinoma in situ |
| 81403 | Adenocarcinoma, NOS |
| 81413 | Scirrhous adenocarcinoma |
| 81443 | Adenocarcinoma, intestinal type |
| 81473 | Basal cell adenocarcinoma |
| 82003 | Adenoid cystic carcinoma |
| 82303 | Solid carcinoma, NOS |
| 82313 | Carcinoma simplex |
| 82502 | Adenocarcinoma in situ, non-mucinous |
| 82503 | Bronchiolo-alveolar adenocarcinoma |
| 82513 | Alveolar adenocarcinoma |
| 82522 | Bronchioalveolar adenocarcinoma in situ |
| 82523 | Bronchiolo-alveolar carcinoma, non-mucinous |
| 82533 | Bronchiolo-alveolar carcinoma, mucinous |
| 82543 | Bronch.-alv. carc., mixed mucin. and non-mucinous |
| 82553 | Adenocarcinoma with mixed subtypes |
| 82603 | Papillary adenocarcinoma, NOS |
| 82633 | Adenocarcinoma in tubulovillous adenoma |
| 83103 | Clear cell adenocarcinoma, NOS |
| 83203 | Granular cell carcinoma |
| 83233 | Mixed cell adenocarcinoma |
| 84303 | Mucoepidermoid carcinoma |
| 84803 | Mucinous adenocarcinoma |
| 84813 | Mucin-producing adenocarcinoma |
| 84903 | Signet ring cell carcinoma |
| 85503 | Acinar cell carcinoma |
| 85603 | Adenosquamous carcinoma |
| 85723 | Adenocarcinoma with spindle cell mataplasia |
| 85743 | Adenocarcinoma with neuroendocrine differen. |
| 85753 | Metaplastic carcinoma, NOS |
| 85763 | Hepatoid adenocarcinoma |
| 88033 | Small cell sarcoma |
| 89403 | Mixed tumor, malignant, NOS |
| 89803 | Carcinosarcoma, NOS |
| 90153 | Mucinous adenocarcinofibroma |

*Codes determined based clinician review and references as below:

Santana-Davila R, Devisetty K, Szabo A, Sparapani R, Arce-Lara C, Gore EM, Moran A, Williams CD, Kelley MJ, Whittle J. Cisplatin and etoposide versus carboplatin and paclitaxel with concurrent radiotherapy for stage III non-small-cell lung cancer: an analysis of Veterans Health Administration data. J Clin Oncol. 2015 Feb 20;33(6):567-74.

**Appendix Table 3a. CPT Codes for Lung Resection**

| **Code** | **Description** |
| --- | --- |
| 32505 | Thoracotomy; with therapeutic wedge resection (eg, mass, nodule), initial |
| 32506 | Thoracotomy; with therapeutic wedge resection (eg, mass or nodule), each additional resection, ipsilateral (List separately in addition to code for primary procedure) |
| 32507 | Thoracotomy; with diagnostic wedge resection followed by anatomic lung resection (List separately in addition to code for primary procedure) |
| 32666 | Thoracoscopy, surgical; with therapeutic wedge resection (eg, mass, nodule), initial unilateral |
| 32667 | Thoracoscopy, surgical; with therapeutic wedge resection (eg, mass or nodule), each additional resection, ipsilateral (List separately in addition to code for primary procedure) |
| 32668 | Thoracoscopy, surgical; with diagnostic wedge resection followed by anatomic lung resection (List separately in addition to code for primary procedure) |
| 32669 | Thoracoscopy, surgical; with removal of a single lung segment (segmentectomy) |
| 32670 | Thoracoscopy, surgical; with removal of two lobes (bilobectomy) |
| 32671 | Thoracoscopy, surgical; with removal of lung (pneumonectomy) |
| 32520 | Resection of lung; with resection of chest wall |
| 32522 | Resection of lung; with reconstruction of chest wall, without prosthesis |
| 32525 | Resection of lung; with major reconstruction of chest wall, with prosthesis |
| 32440 | Removal of lung, pneumonectomy |
| 32442 | Removal of lung, pneumonectomy; with resection of segment of trachea followed by broncho-tracheal anastomosis (sleeve pneumonectomy) |
| 32445 | Removal of lung, pneumonectomy; extrapleural |
| 32480 | Removal of lung, other than pneumonectomy; single lobe (lobectomy) |
| 32482 | Removal of lung, other than pneumonectomy; 2 lobes (bilobectomy) |
| 32484 | Removal of lung, other than pneumonectomy; single segment (segmentectomy) |
| 32486 | Removal of lung, other than pneumonectomy; with circumferential resection of segment of bronchus followed by broncho-bronchial anastomosis (sleeve lobectomy) |
| 32488 | Removal of lung, other than pneumonectomy; with all remaining lung following previous removal of a portion of lung (completion pneumonectomy) |
| 32491 | Removal of lung, other than pneumonectomy; with resection-plication of emphysematous lung(s) (bullous or non-bullous) for lung volume reduction, sternal split or transthoracic approach, includes any pleural procedure, when performed |
| 32501 | Resection and repair of portion of bronchus (bronchoplasty) when performed at time of lobectomy or segmentectomy (List separately in addition to code for primary procedure) |
| 32655 | Thoracoscopy, surgical; with resection-plication of bullae, includes any pleural procedure when performed |
| 32656 | Thoracoscopy, surgical; with parietal pleurectomy |
| 32662 | Thoracoscopy, surgical; with excision of mediastinal cyst, tumor, or mass |
| 32663 | Thoracoscopy, surgical; with lobectomy (single lobe) |
| 32503 | Resection of apical lung tumor (eg, Pancoast tumor), including chest wall resection, rib(s) resection(s), neurovascular dissection, when performed; without chest wall reconstruction(s) |
| 32504 | Resection of apical lung tumor (eg, Pancoast tumor), including chest wall resection, rib(s) resection(s), neurovascular dissection, when performed; with chest wall reconstruction |

**Appendix Table 3b. ICD-9 Procedure Codes for Lung Resection**

| **Code** | **Description** |
| --- | --- |
| 32 | Local excision or destruction of lesion or tissue of bronchus |
| 32.01 | Endoscopic excision or destruction of lesion or tissue of bronchus |
| 32.09 | Other local excision or destruction of lesion or tissue of bronchus |
| 32.1 | Other excision of bronchus |
| 32.2 | Local excision or destruction of lesion or tissue of lung |
| 32.2 | Thoracoscopic excision of lesion or tissue of lung |
| 32.28 | Endoscopic excision or destruction of lesion or tissue of lung |
| 32.29 | Other local excision or destruction of lesion or tissue of lung |
| 32.3 | Segmental resection of lung |
| 32.3 | Thoracoscopic segmental resection of lung |
| 32.39 | Other and unspecified segmental resection of lung |
| 32.4 | Lobectomy of lung |
| 32.41 | Thoracoscopic lobectomy of lung |
| 32.49 | Other lobectomy of lung |
| 32.5 | Pneumonectomy |
| 32.5 | Thoracoscopic pneumonectomy |
| 32.59 | Other and unspecified pneumonectomy |
| 32.6 | Radical dissection of thoracic structures |
| 32.9 | Other excision of lung |

**Appendix Table 3c. ICD-10 Procedure Codes for Lung Resection**

| **Code** | **Description** |
| --- | --- |
| 0BT% | Respiratory System, Resection |
| 0BB% | Respiratory System, Excision |

*Codes based on clinician review and references as below:

Brescia AA, Harrington CA, Mazurek AA, Ward ST, Lee JSJ, Hu HM, Brummett CM,

Waljee JF, Lagisetty PA, Lagisetty KH. Factors Associated With New Persistent

Opioid Usage After Lung Resection. Ann Thorac Surg. 2019 Feb;107(2):363-368.

Lynch JA, Berse B, Rabb M, Mosquin P, Chew R, West SL, Coomer N, Becker D,

Kautter J. Underutilization and disparities in access to EGFR testing among

Medicare patients with lung cancer from 2010 - 2013. BMC Cancer. 2018 Mar

20;18(1):306.

Shirvani SM, Jiang J, Chang JY, Welsh J, Likhacheva A, Buchholz TA, Swisher

SG, Smith BD. Lobectomy, sublobar resection, and stereotactic ablative

radiotherapy for early-stage non-small cell lung cancers in the elderly. JAMA

Surg. 2014 Dec;149(12):1244-53. doi: 10.1001/jamasurg.2014.556.

<https://www.ethicon.com/na/system/files/2018-03/087495-180207_2018_Rmbrsmnt_FS_Thoracic_r5%20%281%29.pdf>

**Appendix Table 4. Stop Codes for VA Oncology Clinics**

| **StopCode** | **StopCodeName** |
| --- | --- |
| 316 | ONCOLOGY/TUMOR |
| 330 | CHEMOTHERAPY PROC. UNIT-MED. |
| 308 | HEMATOLOGY/ONCOLOGY |
| 149 | RADIATION ONCOLOGY |
| 472 | CHEMO RX2 |
| 451 | SURGICAL ONCOLOGY |
| 452 | OSTOMY/ONCOLOGY (LOCAL DSS) |
| 465 | RADIATION THERAPY |
| 471 | CHEMO Rx1 |
| 460 | COLLATERAL ONCOLOGY SUPPORT |
| 476 | TELEPHONE ONCOLOGY |
| 111 | ONCOLOGICAL NUCLEAR MED |
| 431 | CHEMOTHERAPY PROC. UNIT-SURG. |
| 34 | HEMATOLOGY |
| 42 | ONCOLOGY/TUMOR |
| 93 | RADIATION THERAPY |
| 94 | CHEMOTHERAPY |
| 488 | SURG ONCOLOGY |
| 903 | RADIATION THERAPY |
| 904 | CHEMOTHERAPY |

**Appendix Table 5. Chemotherapy Drugs**

| cisplatin |
| --- |
| vinorelbine |
| etoposide |
| gemcitabine |
| docetaxel |
| pemetrexed |
| paclitaxel |
| vinblastine |
| carboplatin |

*Drugs based on clinician review and references as below:

National Comprehensive Cancer Network (NCCN). NCCN Clinical Practice Guidelines in Oncology. Non-Small Cell Lung Cancer Version 4.2018. 2018 Apr 26;National Comprehensive Cancer Network.

**Appendix Table 6. Radiation Therapy Codes**

| **Code Type** | **Code** | **Description** |
| --- | --- | --- |
| CPT4 | 32553 | Placement of interstitial device(s) for radiation therapy guidance (eg, fiducial markers, dosimeter), percutaneous, intra-thoracic, single or multiple |
| CPT4 | 32701 | Thoracic target(s) delineation for stereotactic body radiation therapy (SRS/SBRT), (photon or particle beam), entire course of treatment |
| CPT4 | 55876 | Placement of interstitial device(s) for radiation therapy guidance (eg, fiducial markers, dosimeter), prostate (via needle, any approach), single or multiple |
| CPT4 | 76950 | Ultrasonic guidance for placement of radiation therapy fields |
| CPT4 | 77014 | Computed tomography guidance for placement of radiation therapy fields |
| CPT4 | 77338 | Multi-leaf collimator (MLC) device(s) for intensity modulated radiation therapy (IMRT), design and construction per IMRT plan |
| CPT4 | 77385 | Intensity modulated radiation treatment delivery (IMRT), includes guidance and tracking, when performed; simple |
| CPT4 | 77386 | Intensity modulated radiation treatment delivery (IMRT), includes guidance and tracking, when performed; complex |
| CPT4 | 77387 | Guidance for localization of target volume for delivery of radiation treatment delivery, includes intrafraction tracking, when performed |
| CPT4 | 77399 | Unlisted procedure, medical radiation physics, dosimetry and treatment devices, and special services |
| CPT4 | 77401 | Radiation treatment delivery, superficial and/or ortho voltage, per day |
| CPT4 | 77402 | Radiation treatment delivery, >=1 MeV; simple |
| CPT4 | 77403 | Radiation treatment delivery, single treatment area, single port or parallel opposed ports, simple blocks or no blocks; 6-10 MeV |
| CPT4 | 77404 | Radiation treatment delivery, single treatment area, single port or parallel opposed ports, simple blocks or no blocks; 11-19 MeV |
| CPT4 | 77407 | Radiation treatment delivery, >=1 MeV; intermediate |
| CPT4 | 77408 | Radiation treatment delivery, 2 separate treatment areas, 3 or more ports on a single treatment area, use of multiple blocks; 6-10 MeV |
| CPT4 | 77409 | Radiation treatment delivery, 2 separate treatment areas, 3 or more ports on a single treatment area, use of multiple blocks; 11-19 MeV |
| CPT4 | 77411 | Radiation treatment delivery, 2 separate treatment areas, 3 or more ports on a single treatment area, use of multiple blocks; 20 MeV or greater |
| CPT4 | 77412 | Radiation treatment delivery, >=1 MeV; complex |
| CPT4 | 77413 | Radiation treatment delivery, 3 or more separate treatment areas, custom blocking, tangential ports, wedges, rotational beam, compensators, electron beam; 6-10 MeV |
| CPT4 | 77414 | Radiation treatment delivery, 3 or more separate treatment areas, custom blocking, tangential ports, wedges, rotational beam, compensators, electron beam; 11-19 MeV |
| CPT4 | 77416 | Radiation treatment delivery, 3 or more separate treatment areas, custom blocking, tangential ports, wedges, rotational beam, compensators, electron beam; 20 MeV or greater |
| CPT4 | 77421 | Stereoscopic X-ray guidance for localization of target volume for the delivery of radiation therapy |
| CPT4 | 77763 | Intracavitary radiation source application; complex |
| CPT4 | 77778 | Interstitial radiation source application, complex, includes supervision, handling, loading of radiation source, when performed |
| CPT4 | 77790 | Supervision, handling, loading of radiation source |
| CPT4 | 0197T | Intra-fraction localization and tracking of target or patient motion during delivery of radiation therapy (eg, 3D positional tracking, gating, 3D surface tracking), each fraction of treatment |
| HCPCS | G6001 | Ultrasonic guidance for placement of radiation therapy fields |
| HCPCS | G6002 | Stereoscopic x-ray guidance for localization of target volume for the delivery of radiation therapy |
| HCPCS | G6012 | Radiation treatment delivery,3 or more separate treatment areas, custom blocking, tangential ports, wedges, rotational beam, compensators, electron beam; 6-10 mev |
| HCPCS | G6013 | Radiation treatment delivery,3 or more separate treatment areas, custom blocking, tangential ports, wedges, rotational beam, compensators, electron beam; 11-19 mev |
| HCPCS | G6014 | Radiation treatment delivery,3 or more separate treatment areas, custom blocking, tangential ports, wedges, rotational beam, compensators, electron beam; 20 mev or greater |
| HCPCS | G6017 | Intra-fraction localization and tracking of target or patient motion during delivery of radiation therapy (eg,3d positional tracking, gating, 3d surface tracking), each fraction of treatment |
| ICD10PCS | D0001ZZ | Beam Radiation of Brain using Photons 1 - 10 MeV |
| ICD10PCS | D7051ZZ | Beam Radiation of Thorax Lymphatics using Photons 1 - 10 MeV |
| ICD10PCS | D7052ZZ | Beam Radiation of Thorax Lymphatics using Photons >10 MeV |
| ICD10PCS | DB001ZZ | Beam Radiation of Trachea using Photons 1 - 10 MeV |
| ICD10PCS | DB011ZZ | Beam Radiation of Bronchus using Photons 1 - 10 MeV |
| ICD10PCS | DB012ZZ | Beam Radiation of Bronchus using Photons >10 MeV |
| ICD10PCS | DB020ZZ | Beam Radiation of Lung using Photons <1 MeV |
| ICD10PCS | DB021ZZ | Beam Radiation of Lung using Photons 1 - 10 MeV |
| ICD10PCS | DB022ZZ | Beam Radiation of Lung using Photons >10 MeV |
| ICD10PCS | DB023ZZ | Beam Radiation of Lung using Electrons |
| ICD10PCS | DB024ZZ | Beam Radiation of Lung using Heavy Particles (Protons,Ions) |
| ICD10PCS | DB061ZZ | Beam Radiation of Mediastinum using Photons 1 - 10 MeV |
| ICD10PCS | DB062ZZ | Beam Radiation of Mediastinum using Photons >10 MeV |
| ICD10PCS | DB063ZZ | Beam Radiation of Mediastinum using Electrons |
| ICD10PCS | DB064ZZ | Beam Radiation of Mediastinum using Heavy Particles (Protons,Ions) |
| ICD10PCS | DBY17ZZ | Contact Radiation of Bronchus |
| ICD10PCS | DBY27ZZ | Contact Radiation of Lung |
| ICD10PCS | DBY2FZZ | Plaque Radiation of Lung |
| ICD10PCS | DBY6FZZ | Plaque Radiation of Mediastinum |
| ICD10PCS | DP092ZZ | Beam Radiation of Femur using Photons >10 MeV |
| ICD10PCS | DP0C1ZZ | Beam Radiation of Other Bone using Photons 1 - 10 MeV |
| ICD10PCS | DW011ZZ | Beam Radiation of Head and Neck using Photons 1 - 10 MeV |
| ICD10PCS | DW021ZZ | Beam Radiation of Chest using Photons 1 - 10 MeV |
| ICD10PCS | DW022ZZ | Beam Radiation of Chest using Photons >10 MeV |
| ICD10PCS | DWY27ZZ | Contact Radiation of Chest |
| ICD9Proc | 92.22 | Orthovoltage radiation |
| ICD9Proc | 92.26 | Teleradiotherapy of other particulate radiation |

*Codes based on pulling codes related to radiation within VA system, clinician review, and references as below:

<https://aimspecialtyhealth.com/guidelines/PDFs/2019/Jan28/AIM_Guidelines_RadiationOncology.pdf>

<http://www.hcsrn.org/crn/en/RESEARCH/LookupTables/>

**Appendix Table 7. Variation in Treatment Rates Across 108 Medical Centers**

|  | **Mean (standard deviation)** | **Median**  **(25^th^ – 75^th^ percentile)** |
| --- | --- | --- |
| **Concurrent CRT** | 45% (18%) | 47% (34-56%) |
| **Sequential CRT** | 12% (10%) | 10% (4-18%) |
| **Radiotherapy Only** | 11% (10%) | 10% (4-15%) |
| **Chemotherapy Only** | 16% (14%) | 13% (7-20%) |
| **Neither** | 16% (11%) | 15% (10-20%) |

**Appendix Table 8. Treatment Rates Based on Expanding Initial Treatment Time Window from 120 days to 180 days**

| **Treatment** | **Number of Patients (%)** |
| --- | --- |
| **CCRT (concurrent)** | 1964 (48%) |
| **SCRT (sequential)** | 631 (16%) |
| **RT only** | 458 (11%) |
| **CT only** | 501 (12%) |
| **Neither** | 500 (12%) |

**Appendix Table 9. Treatment Rates Based on Varying Time Window Between CT and RT to Define CCRT**

| **Treatment** | **Number of Patients (%)** | | | |
| --- | --- | --- | --- | --- |
|  | **cCRT defined as CT and RT starting within +/- 7 days** | **cCRT defined as CT and RT starting within +/- 14 days** | **cCRT defined as CT and RT starting within +/- 21 days** | **cCRT defined as CT and RT starting within +/- 30 days** |
| **cCRT (concurrent)** | 1449 (36%) | 1893 (47%) | 2106 (52%) | 2225 (55%) |
| **sCRT (sequential)** | 958 (24%) | 514 (13%) | 301 (7%) | 182 (4%) |
| **RT only** | 466 (11%) | | | |
| **CT only** | 541 (13%) | | | |
| **Neither** | 640 (16%) | | | |

**Appendix Table 10. Reasons for Not Receiving CCRT per Chart Review**

| **Reason for not receiving CCRT** | **Count** |
| --- | --- |
| Not a candidate | 60 |
| Too weak or low performance status | 24 |
| Disease too extensive or lung nodules too far apart for radiation therapy | 14 |
| Comorbidities | + |
| Further information not provided | + |
| Disease progressed or multiple cancers | + |
| Age | + |
| Patient refused | 25 |
| **Total** | **85** |

+ Cell size <11 suppressed per VA policy

**Appendix Figure 1. Total and Annual Treatment Rates in the Subgroup Seen at Medical Centers with Radiation Therapy (n=2,067)**

**Appendix Figure 2. Treatment Rates by Census Region in the Subgroup Seen at Medical Centers with Radiation Therapy (n=2,067)**

**Appendix Figure 3. Boxplot of Variation in Treatment Rates Across 38* Medical Centers that Provide Radiation Therapy**

**
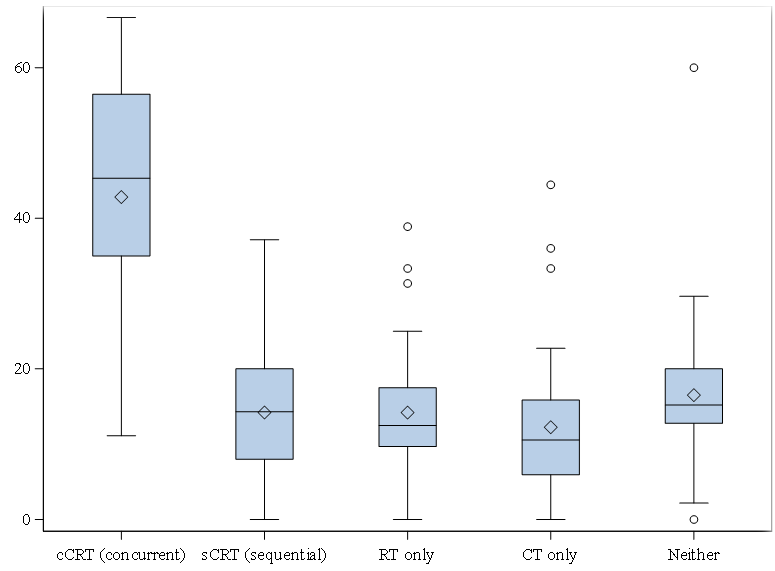
**

*There are actually 39 medical centers that provide radiation therapy, but our study sample was not diagnosed with stage III NSCLC at one of these centers.
